# Insight Into Inflammasome Signaling: Implications for *Toxoplasma gondii* Infection

**DOI:** 10.3389/fimmu.2020.583193

**Published:** 2020-12-16

**Authors:** Yang Wang, Jinjin Zhu, Yuanyuan Cao, Jilong Shen, Li Yu

**Affiliations:** Department of Microbiology and Parasitology, Anhui Provincial Laboratory of Microbiology and Parasitology, Anhui Provincial Laboratory of Zoonoses of High Institutions, School of Basic Medical Sciences, Anhui Medical University, Hefei, China

**Keywords:** *Toxoplasma gondii*, NLRP1, NLRP3, AIM2, inflammasome, infection

## Abstract

Inflammasomes are multimeric protein complexes regulating the innate immune response to invading pathogens or stress stimuli. Recent studies have reported that nucleotide-binding leucine-rich repeat-containing (NLRs) proteins and DNA sensor absent in melanoma 2 (AIM2) serve as inflammasome sentinels, whose stimulation leads to the proteolytic activation of caspase-1, proinflammatory cytokine secretion, and pyroptotic cell death. Toxoplasma gondii, an obligate intracellular parasite of phylum Apicomplexans, is reportedly involved in NLRP1, NLRP3 and AIM2 inflammasomes activation; however, mechanistic evidence regarding the activation of these complexes is preliminary. This review describes the current understanding of inflammasome signaling in rodent and human models of T. gondii infection.

## Introduction

Toxoplasma gondii is an obligate intracellular protozoan parasite potentially invading all nucleated cells of warm-blooded vertebrates, infecting approximately one-third of the human population worldwide. In immunocompetent individuals, a generally asymptomatic acute infection is followed by the onset of a quiescent bradyzoite parasite stage, leading to long-term chronic infections. However, in immunosuppressed individuals, the bradyzoites in the cyst become proliferating tachyzoites and can induce marked tissue damage, causing severe toxoplasmosis and distant dissemination. Previous studies have reported that multiple genetic types of T. gondii are distributed worldwide, of which types I, II and III are the classical strains in North America and Europe (1). Furthermore, *T. gondii* strains are much more genetically diverse in South America; however, most parasites in East Asia fall within one nonclassical lineage: Chinese 1 (2). Among laboratory mice, excluding wild mice or other intermediate hosts (3), type I parasites kill the host in the acute phase of the infection, possibly due to hyperinflammation-inducible cytokine storm and uncontrolled parasite dissemination. Type II and III strains typically exhibit less virulence and may even be avirulent.

**Figure 1:**
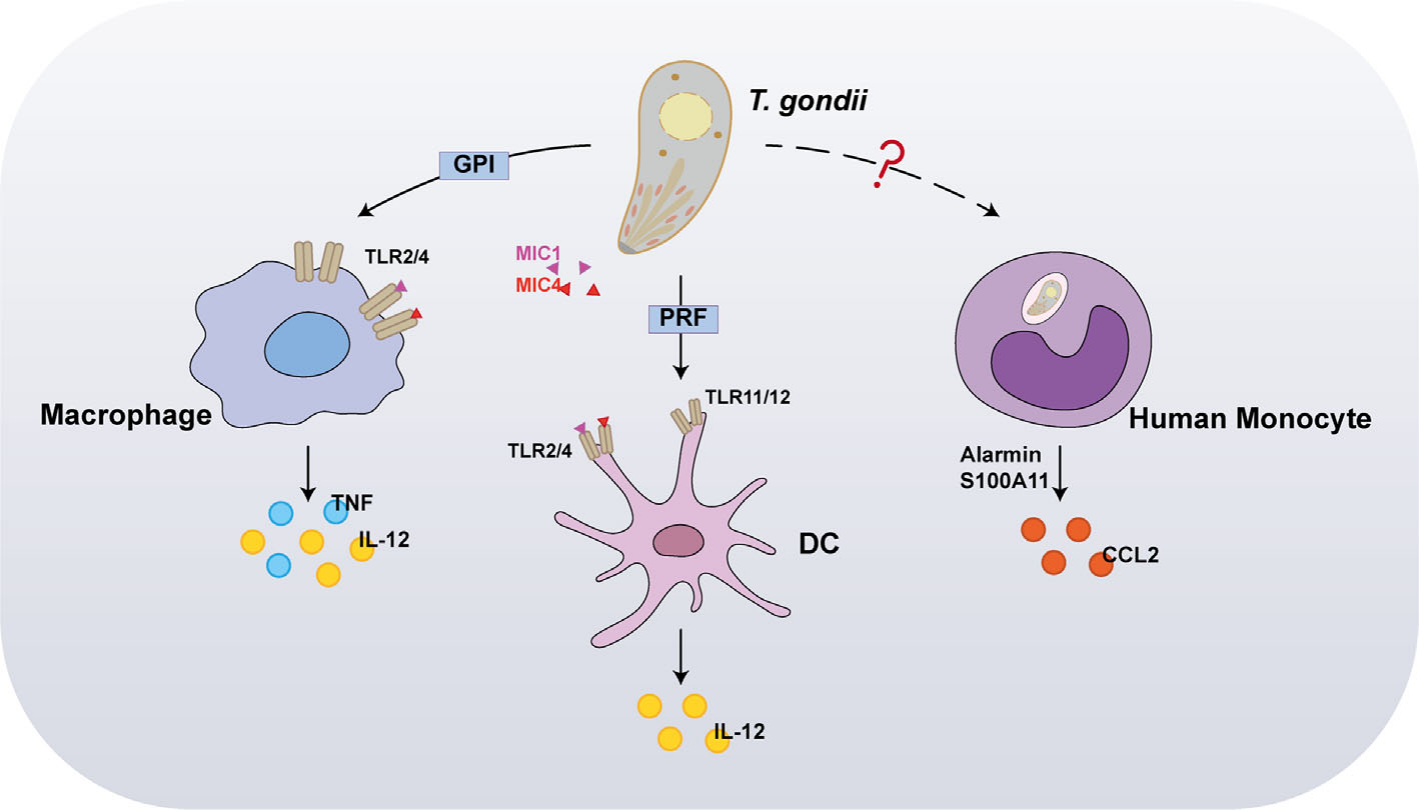
Recognition of *Toxoplasma gondii* by innate immunity. In the early stages of infection, the first host cells to respond are dendritic cells (DCs), monocytes, and macrophages. Interaction between *T. gondii* profilin and Toll-like receptor 11 (TLR11) is important for host production of interleukin-12 (IL-12). In addition to stimulating IL-12 production, macrophages also induce tumor necrosis factor (TNF) in response to TLR2 and TLR4-mediated detection of glycosylphosphatidylinositol (GPI). Furthermore, the interaction between *T. gondii* microneme protein (MIC) 1/4 with TLR2/4 N-glycans triggers IL-12 secretion in both DCs and macrophages. Alarmin S100A11, a damage-associated molecule (DAM) released from infected cells, was recently reported to stimulate chemokine CCL2 secretion in a RAGE-dependent manner against *T. gondii* infection on human monocytes.

Pioneer studies have reported that, at the early stage of T. gondii infections, dendritic cells (DCs), monocytes, and macrophages are the initial host responders. Innate immune cells use pattern recognition receptors (PRRs) to sense molecules associated with invaders, called pathogen-associated molecular patterns (PAMPs) (**Figure 1**). Host PRRs are involved in pathogen recognition, including membrane-bound Toll-like receptors (TLRs), cytosolic nucleotide-binding leucine-rich repeat-containing (NLRs) proteins, and the cytosolic DNA sensor absent in melanoma 2 (AIM2), which play vital physiological roles including antigen presentation, cell death, and cytokine secretion (4, 5). Myeloid differentiation primary response 88 (MyD88), a primary adapter protein, influences the function of multiple TLRs and regulates IL-12 secretion from DCs, macrophages, and neutrophils in response to a T. gondii infection, implying that TLRs are involved in microbial recognition. Thus far, numerous studies have reported that specific TLRs participate in T. gondii recognition, including TLR2, TLR4, TLR7, TLR9, TLR11, and TLR12. Among these, TLR2 and TLR4 participate in recognizing parasites by macrophages via interaction with glycosylphosphatidylinositol (GPI) anchors protein of T. gondii, whereas TLR7 and TLR9 detect the parasite’s nucleic acids (6). In contrast to GPI, profilin (PRF), another T. gondii PAMP, is mediated through recognition by TLR11 and TLR12 in DCs, since it is highly susceptible to T. gondii infections in mice deficient in UNC93B1, a chaperon for these two endosomal nucleic acid-sensing TLRs (7, 8). Concurrently, both TLR11^–/–^ and TLR12^–/–^ mice are highly susceptible to infection, displaying severe deficits in IL-12 and IFN-γ production. Therefore, TLR11 and TLR12 have been considered major players in the recognition of T. gondii by DCs. Furthermore, recent studies have reported that the interaction between T. gondii surface adhesins microneme protein (MIC) 1/4 with TLR2/4 N-glycans triggers IL-12 secretion in both DCs and macrophages (9, 10). Hence, further studies are required to investigate the function of TLR signaling in proinflammatory cytokine production and host protection after T. gondii infection in innate immune cells.

In marked contrast to murine cells, in human cells, TLR11/TLR12 was reportedly do not function in T. gondii PAMPs recognition, owing to a lack of functional TLR11 and TLR12 genes. Recently, alarmin S100A11, a damage-associated molecule (DAM) released from infected cells, was recently reported to stimulate chemokine CCL2 secretion in a RAGE-dependent manner against T. gondii infection on monocytes (11); however, it remains unknown whether the innate immune sensor directly interacts with T. gondii PAMPs. Furthermore, inflammasome activation is partly required for T. gondii recognition in both murine and human cells. NLRPs are cytosolic proteins, which are poised to respond specifically to protozoan parasites. Indeed, NLRP1, NLRP3, and AIM2 form inflammasome complexes with the effector caspase in response to a *T. gondii* infection.

## Response of NLRP1, NLRP3, and AIM2 Inflammasomes to Pathogen-Associated Triggers

**Figure 2:**
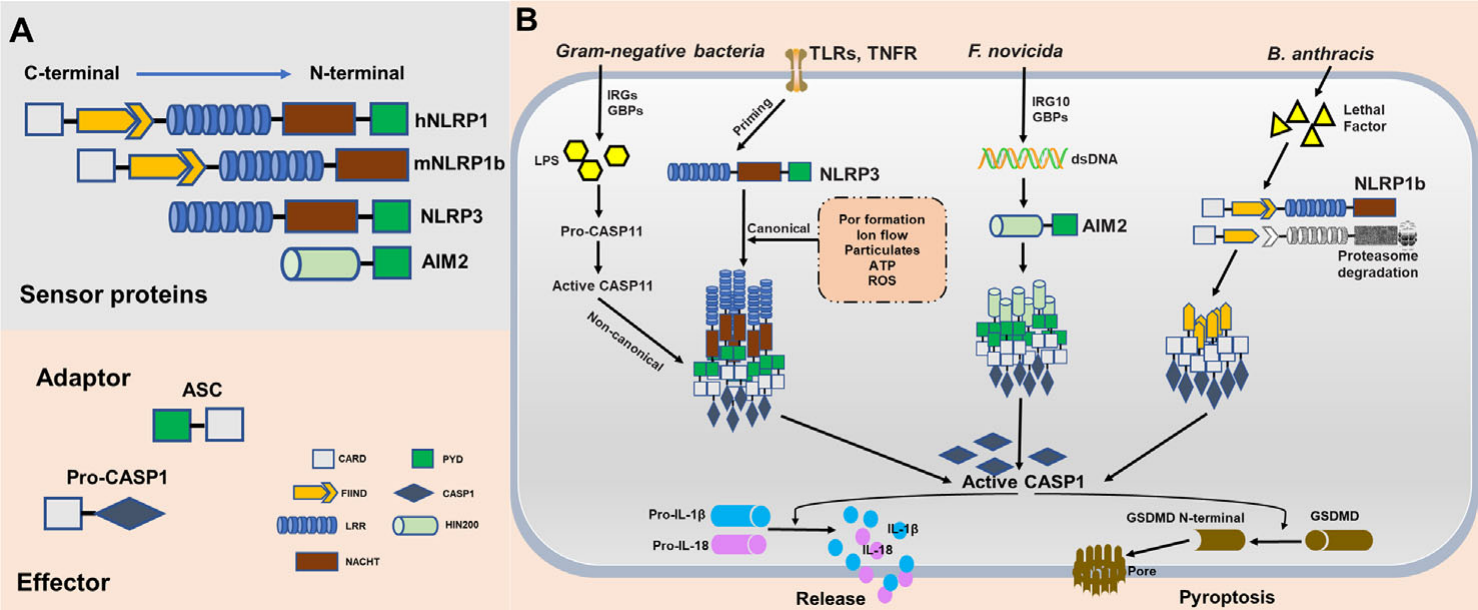
Molecular composition and activation of the NLRP1, NLRP3, and absent in melanoma 2 (AIM2), inflammasomes. **(A)** Inflammasome sensor proteins containing a leucine-rich-repeat domain (LRR), a nucleotide-binding domain (NACHT), a HIN-200 domain, a caspase-activation and recruitment domain (CARD) and/or a pyrin domain (PYD). PYD of hNLRP1, NLRP3, and AIM2 potentially recruit adaptor protein apoptosis-associated speck-like protein containing a CARD (ASC) to mediate CARD–CARD interactions with the effector caspase. As opposed to humans, mouse NLRP1b does not encode PYD. However, the CARD of mNLRP1b can interact directly with caspase without the ASC. **(B)** NLRP1b, NLRP3, and AIM2 are activated in response to various stimuli and/or pathogens. Activated NLRP1b, NLRP3, and AIM2 recruits and activates caspase-1 to initiate pyroptosis through gasdermin D (GSDMD) cleavage. Caspase-1 also releases IL-1β and IL-18 through pro-interleukin (IL)-1β and pro-IL-18 cleavage.

Inflammasomes are cytosolic multimeric protein complexes responding to microbial molecules or stress signals, which defend the host against pathogens. In general, sensor proteins, including NLRPs or AIM2, detect PAMPs or danger-associated molecular patterns (DAMPs) in the cytoplasm to initiate oligomerization, facilitating the recruitment of the adapter apoptosis-associated speck-like protein containing a caspase-activation and recruitment domain (CARD) (ASC) (12). The CARD of ASC or the CARD of the sensor protein recruits inflammatory caspase-1 monomers *via* homotypic protein domain interactions. The inflammasome platform leads to the dimerization of caspase-1, thus activating protease function, and caspase-1 subsequently triggers the release of mature interleukin-1β (IL-1β) and IL-18 (13). Moreover, activated caspase-1 cleaves gasdermin D (GSDMD) into its N-terminal form, yielding a pore-forming fragment that targets the plasma membrane, leading to pyroptosis (14). All proteins of the NLR family contain a central nucleotide-binding domain (NACHT), which comprises an NBD, helical domain 1 (HD1), winged-helix domain (WHD) and helical domain 2 (HD2); a short C-terminal leucine-rich repeat (LRR) domain; and most have a variable N-terminal domain. NLRP3, the most studied and characterized inflammasome, contains an N-terminal effector domain pyrin domain (PYD). The PYD of NLRP3 interacts with the PYD of ASC, and the CARD domain of ASC interacts with the CARD domain of effector caspases (15). The mitotic kinase NEK7 was recently reported to be required for the stimulus-dependent NLRP3 activation by building oligomerization (16). Human NLRP1 also contains a domain termed function to find domain (FIIND), which is an autolytic cleavage domain, and a C-terminal CARD domain. In contrast, mouse NLRP1 (mouse genome encodes for three NLRP1 paralogs, a-c), which does not contain a PYD domain, directly interacts with a caspase (17). Therefore, the adapter protein ASC is necessary for activating the NLRP3 inflammasome to induce caspase cleavage and is not necessary for NLRP1. Furthermore, ASC contributes to NLRP-independent inflammasome activation. AIM2 contains a nuclear localization (HIN) 200 domain responsible for cytosolic double-strands DNA (dsDNA) binding and a PYD domain that interacts with ASC and subsequently activates the AIM2 inflammasome (18) (**Figure 2A**).

Although NLRP1, NLRP3, and AIM2 together contribute to host defense, they are distinct in terms of ligand binding, complex composition, and activation mechanisms. NLRP1 was the first reported NLR to form an inflammasome complex; however, its activation mechanism remains unclear (19). mNLRP1b is activated through direct cleavage by the lethal factor (LF) protease secreted by *Bacillus anthracis*. An underlying activation mechanism, termed “functional degradation”, has been proposed. The self-cleavage of NLRP1b within its FIIND(ZU5+UPA) domain is required for subsequent inflammasome activation. Pathogenic trigger, such as LF cleaves the N-terminal fragment of NLRP1b, leading to ubiquitination and degradation of the unstable fragment by the E3 ubiquitin ligase UBR2. This functional degradation event releases autoinhibition of the CARD-containing C-terminal fragment of the protein, permitting it to oligomerize and form an activated inflammasome platforms (20–23). Consistent with this model, another pathogenic enzyme named IpaH7.8, a *Shigella flexneri* ubiquitin ligase secreted effector, was found to induce NLRP1b degradation and activation. However, it remains unclear whether this functional degradation event triggers NLRP1b activation in *T. gondii*. Currently, the mechanism underlying the activation of the NLRP3 inflammasome remains unclear. Initial priming and activation are required for complete activation of NLRP3. In both human and murine cells, the initial priming step requires recognition of PAMPs by PRRs to activate *NLRP3* expression, ensuring that the cell is ready to respond to NLRP3 triggers. Numerous triggers, including ion flow (potassium efflux and calcium influx), ATP, lysosomal disruption by crystalline particulates, and reactive oxygen species (24–26), have been proposed to activate canonical NLRP3 signaling, implying numerous potential mechanism of action for this inflammasome. For instance, *Leishmania* induces the generation of reactive oxygen species (ROS) to activate the NLRP3 inﬂammasome, and the major glycoconjugate present on the surface of this parasite triggers caspase-11 dependent NLRP3 inﬂammasome activation (27, 28). Similarly, a *Neospora caninum-*induced potassium efflux helps activate the NLRP3 inﬂammasome (29). Furthermore, NLRP3 activation by gram-negative bacteria or cytosolic LPS has been referred to as non-canonical signaling. This activation mechanism dependents on murine caspase-11, human caspase-4, and caspase-5. Stimulation by LPS and caspase-4/5/11 facilitates the cleavage of GSDMD, leading to pyroptosis (30, 31). The mechanisms of action of the AIM2 inflammasome are less clear than those of the NLRP1 and NLRP3 inflammasome. Several groups independently identify guanylate-binding proteins (GBPs) and IRGB10 as essential mediators of AIM2 activation in *Francisella novicida* infections (32). Mechanistically, these IFN-inducible GTPases target cytosolic pathogens and promote bacteriolysis, liberating bacterial dsDNA into the cytosol, which is then recognized by the DNA-binding protein AIM2 (33, 34) (**Figure 2B**).

## Functions of Inflammasomes in Rodent Models of *T. gondii* Infection

T. gondii infects nearly all nucleated cell types, exhibiting an extremely broad host range. Compared with mice, humans and rats are markedly resistant to T. gondii infection, generally having a subclinical chronic infection. Lewis rats, however, can clear the T. gondii completely, thus inhibiting cysts formation in the host brain and muscle tissues. T. gondii resistance is partially mediated by Toxo1, a major locus of control on chromosome 10 containing Nlrp1 (35). Accordingly, SNP, whole transcriptome and functional studies have identified T. gondii as an activator of the NLRP1 inflammasome in rat macrophages (36, 37). Bone-marrow-derived macrophages (BMDMs) extract from Lewis and spontaneously hypertensive (SHR) rats, both expressing Nlrp1 allele 5, displayed pyroptosis upon stimulation by T. gondii. In contrast, T. gondii induced markedly lesser pyroptosis in Brown Norway (BN), Sprague Dawley (SD), and Fischer (CDF) rat BMDMs expressing Nlrp1 allele 1 or allele 2 (36–38). These T. gondii-resistant rats have T. gondii-sensitive macrophages, indicating a protective role of macrophage pyroptosis upon parasite infection. It should be emphasized that rat Nlrp1 alleles that confer susceptibility to T gondii are the opposite of those conferring LT susceptibility (38, 39).

**Figure 3:**
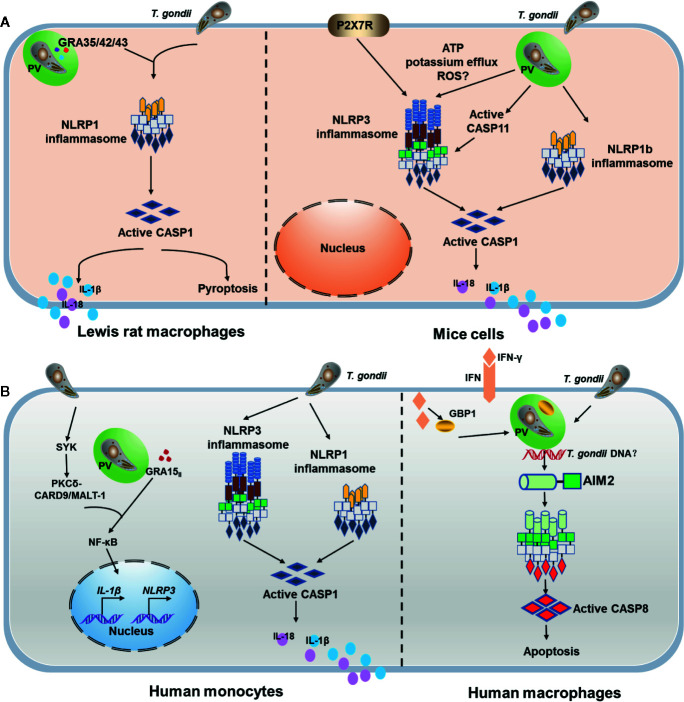
Role of the inflammasome complex in modulating immune responses in a *Toxoplasma gondii* infection. **(A)** *T. gondii*-mediated NLRP1 inflammasome activation in Lewis rat bone-marrow-derived macrophages (BMDMs), subsequently leads to the generation of the biologically active IL-1β and IL-18 cytokines and cell pyroptosis. *T. gondii* dense granule proteins GRA35, GRA42, and GRA43 are involved in NLRP1 inflammasome activation. In murine cells, multiple mechanisms including potassium K+ efflux, ATP, reactive oxygen species (ROS), and CASP11 are involved in NLRP3 activation in a *T. gondii* infection. Furthermore, NLRP1b is activated in parasitic infections, albeit through unclear mechanisms. **(B)** *T. gondii* infection activates both NLRP1 and NLRP3 inflammasomes in human monocytic cells. *T. gondii* induces a Syk-CARD9/MALT-1-NF-κB signaling pathway and activates the NLRP3 inflammasome for IL-1β release, and GRA15 directly activates NF-κB. Recent studies have reported that in human macrophages, IFN-γ impels guanylate binding protein 1 (GBP1) to mediate cell apoptosis *via* the absent in melanoma (AIM2) inflammasome, ASC, and caspase-8 in a *T. gondii* infection.

The NLRP3 inflammasome is dispensable for T. gondii infection in rats, in contrast, both NLRP1 and NLRP3 are important for controlling T. gondii in mice (40, 41). Mice are more susceptible to T. gondii infection than rats. T. gondii induces undetectable or far less cell pyroptosis in mouse BMDMs than in rat BMDMs. Previous investigation of caspase-11 function focused on its LPS sensing abilities, which proved to be a powerful trigger for signal transduction in non-canonical inﬂammasome (42). One study demonstrated that caspase-11 functions to protect the host by enhancing inﬂammation during the early phase of infection (43). In mouse macrophages, activation of caspase-11 requires the lysis of vacuole-bearing pathogens by GBPs during infections with gram-negative bacteria (44). When considering type I T. gondii in mice, lethal overproduction of Th1 cytokines, including IL-1β and IL-18, may be implicated in inflammasome activation (45). Moreover, the deletion of mitogen-activated protein kinase 1 (MAPK1) and MAPK2 in type I parasites suppresses NLRP1/3 inflammasome activation, as opposed to wide-type parasites, but the virulence in mice decreased (46). The possible reason causes this discrepancy about the functions of NLRP3 inflammasome in the host against *T. gondii* is that ΔMAPK1 and ΔMAPK2 parasites caused high levels of IFN-β, resulting in the inhibition of inflammasome activation (47), and decreased virulence of parasite may partly attribute to the importance of type I IFN in control of toxoplasmosis as recently reported (48). Nonetheless, the mechanism underlying NLRP1 activation in response to T. gondii infection remains unclear in mice and rats. Recent studies have investigated the mechanism underlying NLRP3 activation in T. gondii-infected mouse macrophages. P2X_7_R, a proinflammatory receptor, was found to be involved in canonical NLRP3 inflammasome activation depending on extracellular ATP (eATP) and thus mediate T. gondii control (49) (**Figure 3A**). In addition to NLRP1/3, NOD2 plays a crucial role in the host adaptive response to *T. gondii*, regulating T cell immune responses. One of the premiere studies on *Toxoplasma* and NLRs reported that NOD2 provides an intrinsic signal in T cells, contributing to the generation of immunity *via* protective T helper cells (Th) 1 against *T. gondii* infection (50). Nod2^-/-^ mice reportedly failed to mount an effective adaptive response, with impaired production of IL-2 and nuclear accumulation of the transcriptional factor c-Rel. Furthermore, it should be mentioned here that NOD2 may also bind to NLRP1 or NLRP3, thereby participating in IL-1β secretion (51–54). Accordingly, further studies are required to investigate the role of NLRP1, NLRP3, and other NLR proteins in *T. gondii* infection.

IFN-γ is the major mediator of resistance to *T. gondii* infection and is essential for generating various antimicrobial activities in numerous cell types, especially in murine cells. These activities involve either direct destruction of the parasitophorous vacuole membranes (PVMs, targeted by IFN-γ inducible GTPases, IRGs, and GBPs), production of NO and ROS, acidification of the intravacuolar environment, or the activation of cell death upon infection (55–57). IL-18 and IL-1β are both required for IFN-γ-mediated immune responses. Therefore, inflammasome activation is expected to potentially affect IFN-γ production and host resistance to *T. gondii.* Concurrently, a previous study recently reported that in the absence of TLR11, inflammasome activation is critical for maintaining CD4^+^ T cell-derived IFN-γ production and mouse resistance to *T. gondii* strain ME49 (58). However, the role of the inflammasome is dispensable and limited when TLR11 remains functional, due to TLR11-dependent IL-12 production is sufficient to generate Th1 immunity to the parasite without inflammasome dependent release of IL-18. Furthermore, Angel K. Kongsomboonvech et al. recently reported that Naïve CD8 T cell IFN-γ responses to *T. gondii* require the inflammasome-independent NLRP3 pathway and rhoptry proteins (ROP) 5 (59). This paper was identified that the NLRP3 regulates T cell function and underscore the need for NLRP3-activating adjuvants in vaccines targeted at inducing CD8 T cell-derived IFN-γ responses to *T. gondii*. Therefore, further experiments will have to investigate the crosstalk between T cell function with the inflammasome pathway.

## The Interplay Between Inflammasomes and *T. gondii* in Human Cells

Because TLR11 is a pseudogene and TLR12 is not expressed in humans, it remains unclear whether inflammasome activation has more pronounced effects in human cells than in murine cells. Moreover, *T. gondii*-induced inflammasome activation varies among different human cell types (**Figure 3B**). A previous study on hNLRP1 activation in toxoplasmosis initially suggested that human susceptibility to congenital toxoplasmosis was mapped to an allele in the NLRP1 gene locus, suggesting a role of the hNLRP1 inflammasome in the human immune response to *T. gondii* (60). In *T. gondii*-infected human monocytic cells, siRNA-mediated silencing of hNLRP1 directly influences cell death, parasite replication, and inflammatory cytokine production. Another study reported that active *T. gondii* invasion stimulated IL-1β secretion in a manner dependent on ASC and caspase-1, and on strain-specific virulence factor GRA15_II_ (an NF−κB activator) (61). Moreover, this study shows that NLRP3 is an inflammasome sensor for *T. gondii*-infected primary human monocytes, resulting in rapid IL-1β release from cells (61). Potassium efflux contributed to this phenomenon, since IL-1β production was significantly reduced in monocytes after extracellular potassium treatment. Furthermore, syk, a spleen tyrosine kinase, participates in NLRP3 activation during fungal and viral infection and is critical IL-1β release from infected primary monocytes (62). Syk phosphorylation was also detected during T. gondii infection, activating the downstream PKCδ-CARD9/MALT-1 pathway and subsequently leading to intranuclear NF-κB translocation, which in turn promotes the expression of *IL1B and NLRP3* (63). Notably, IL-1β production was not altered in GSDMD-knockout THP-1 cells, indicating that IL-1β, which is released from *T. gondii*-infected human monocytes, is independent of GSDMD cleavage and cell pyroptosis in THP-1 cells. Interestingly, unlike in T. gondii-infected primary human monocytes, *T. gondii*-infected human macrophages did not secrete IL-1β or harbor activated NLRP3. Fisch et al. recently provided novel insights into the mechanisms underlying inflammasome activation by type I and type II T. gondii strains in human macrophages. They found *T. gondii* induces an atypical apoptosis pathway involving the AIM2 and promotes apoptosis via ASC and Caspase-8. Interestingly, this phenomenon depends on IFN-γ-stimulated human GBP1 (64). In human small intestinal epithelial cells, P2X7R/NLRP3 pathway was also found to be activated and play important roles in IL-1β secretion and inhibition of T. gondii proliferation (65). Furthermore, T. gondii evades neutrophil-mediated defense by impairing the production of IL-1β and inhibiting the activation of the NLRP3 inflammasome, while facilitating the propagation of the parasite (66). It remains unknown whether the inflammasome is activated in DCs in human and murine cells.

## Effectors of *T. gondii* Modulate the Inflammasome Complex

A successful T. gondii infection requires a delicate balance between host immune responses and the parasite immune evasion, leading to both the host and parasite survival. From another perspective, T. gondii takes advantage of the NLRP3 inflammasome activation, potentially compromising host mechanisms of parasite control. A previous study reported that, T. gondii-infected human monocytes induce IL-1β production in a manner dependent on T. gondii effector protein GRA15 and NLRP3 inflammasome (61). IL-1β combined with IFN-γ robustly stimulates inducible nitric oxide synthase (iNOS) expression. Consequently, indole 2,3-dioxygenase 1 (IDO1) production is severely impaired and facilitates parasite growth in human monocytes (67). GRA7 of T. gondii was previously found to induce expression of pro-inflammatory cytokine genes including IL-1β in macrophages (68). Moreover, it was recently found that it serves as a substrate of PKCα and the recombinant N-terminal -GRA7 interacted with the PYD domain of ASC to promote anti-mycobacterial host defense (69). Whether this effector binds to ASC and induce the expression of pro-inflammatory cytokine in host cells infected with *T. gondii* needs further confirmed. Three other dense granule proteins (GRA35, GRA42, and GRA43) were found to be required for inducing pyroptosis and IL-1β secretion in Lewis rat BMDMs (70). It should be emphasized that T. gondii GRAs are involved in NLRP1 inflammasome activation, albeit the mechanism underlying has not been extensively studied. Furthermore, the T. gondii redox enzyme peroxiredoxin (rTgPrx) was reported to promote alternatively activated macrophage polarization, increasing IL-10 release, while simultaneously impairing caspase-1 activation and IL-1β secretion (71).

## Conclusion and Perspectives

Inflammasome activation can be triggered as a protective host response in the process of pathogen infection. NLRP1, NLRP3 and AIM2 inflammasomes have been identified to participate in the immune response during *T. gondii* infection; however, whether other inflammasomes, e.g., pyrin or newly emerged CARD8 are involved in *T. gondii* infection is required further investigation. As one of the most successful intracellular parasites, *Toxoplasma* develops a series of strategies to fight against host defense. How the parasite fights against inflammasome pathway is still a mystery. Overall, studies on the roles of inflammasomes during *T. gondii* infection are still in their infancy, and their exact roles and activation mechanisms among different cell types remain unknown. Further studies are required to investigate how *T. gondii* is resistant to pyroptosis, and whether the GSDMD plays a role in toxoplasmosis? Although numerous factors including eATP, ROS and potassium efflux are reportedly involved in inflammasome activation, the precise *T. gondii -*derived molecules initiating this process remain unknown. Moreover, AIM2 function as a DNA sensor has been extensively described in virus infection. However, T. gondii is a unique intracellular pathogen different from virus, and it resides within a non-fusogenic parasitophorous vacuole (PV), providing a physical niche for the parasite to shield from intracellular cytoplasmic defense mechanisms. The IFN-γ-inducible GTPases including IRGs and GBPs are essential to disrupt the PV membrane, thereby releasing parasite DNA into the cytoplasm. Are these GTPases involved in AIM2 inflammasome pathway? How inflammasomes crosstalk with interferon-inducible GTPase-mediated host defense? To address these questions, further researches are needed in future. A comprehensive understanding of the mechanisms underlying inflammasome regulation in a *T. gondii* infection will help us strengthen the knowledge that how the host immune responses to this parasite.

## Data Availability Statement

The original contributions presented in the study are included in the article/supplementary material. Further inquiries can be directed to the corresponding authors.

## Author Contributions

LY and YW designed the work. YW and JZ drafted the article and diagramming; YC, JS, and LY did critical revision of the article. All authors contributed to the article and approved the submitted version.

## Funding

This work was supported in part by the National Key R&D Program of China (2017YFD0500400 to LY), National Natural Science Foundation of China (82072304, 81871671 and 81572022 to LY, 81802003 to YC), Outstanding Young Scholars Financial Support of Anhui Medical University (0113014104 to LY), and promotion plan of basic and clinical cooperative research in Anhui Medical University (2019xkjT023 to LY).

## Conflict of Interest

The authors declare that the research was conducted in the absence of any commercial or financial relationships that could be construed as a potential conflict of interest.
